# Appointment of Delegates to National and State Conventions

**Published:** 1848-03

**Authors:** Jno. J. Reese

**Affiliations:** Secretary


### RECORD OF MEDICAL SCIENCE.

### PHILADELPHIA MEDICAL SOCIETY.

*Appointment of Delegates.*

Dr. Huston,

*Dear Sir,*—I enclose you for publication in the Examiner, the lists of the delegates of the Philadelphia Medical Society to the National Medical Association, and to the State Convention, elected February 5th, 1848.

*To the National Medical Association,*—Drs. B. H, Coates, C. Morris, Bell, Bridges, Ashmead, Reese, Emerson, Warrington, I. Parrish, and West.

*To the State Convention,*—Drs. Emerson, Bell, B. H. Coates, Norris, H. H. Smith, Rutter, Shallcross, Bond, R. Paul, and I. Parrish.

Very truly yours,

*Feb.* 16*th*, 1848. Jno. J. Reese, Secretary.

